# Resolution of Cochlear Inflammation: Novel Target for Preventing or Ameliorating Drug-, Noise- and Age-related Hearing Loss

**DOI:** 10.3389/fncel.2017.00192

**Published:** 2017-07-07

**Authors:** Gilda M. Kalinec, Gwen Lomberk, Raul A. Urrutia, Federico Kalinec

**Affiliations:** ^1^Laboratory of Auditory Cell Biology, Department of Head and Neck Surgery, David Geffen School of Medicine, University of CaliforniaLos Angeles, Los Angeles, CA, United States; ^2^Epigenetics and Chromatin Dynamics Laboratory, Translational Epigenomic Program, Center for Individualized Medicine (CIM) Mayo ClinicRochester, MN, United States

**Keywords:** drug-induced hearing loss, noise-induced hearing loss, age-related hearing loss, inflammation, resolution of inflammation, lipid mediators, annexin A1, galectin

## Abstract

A significant number of studies support the idea that inflammatory responses are intimately associated with drug-, noise- and age-related hearing loss (DRHL, NRHL and ARHL). Consequently, several clinical strategies aimed at reducing auditory dysfunction by preventing inflammation are currently under intense scrutiny. Inflammation, however, is a normal adaptive response aimed at restoring tissue functionality and homeostasis after infection, tissue injury and even stress under sterile conditions, and suppressing it could have unintended negative consequences. Therefore, an appropriate approach to prevent or ameliorate DRHL, NRHL and ARHL should involve improving the resolution of the inflammatory process in the cochlea rather than inhibiting this phenomenon. The resolution of inflammation is not a passive response but rather an active, highly controlled and coordinated process. Inflammation by itself produces specialized pro-resolving mediators with critical functions, including essential fatty acid derivatives (lipoxins, resolvins, protectins and maresins), proteins and peptides such as annexin A1 and galectins, purines (adenosine), gaseous mediators (NO, H_2_S and CO), as well as neuromodulators like acetylcholine and netrin-1. In this review article, we describe recent advances in the understanding of the resolution phase of inflammation and highlight therapeutic strategies that might be useful in preventing inflammation-induced cochlear damage. In particular, we emphasize beneficial approaches that have been tested in pre-clinical models of inflammatory responses induced by recognized ototoxic drugs such as cisplatin and aminoglycoside antibiotics. Since these studies suggest that improving the resolution process could be useful for the prevention of inflammation-associated diseases in humans, we discuss the potential application of similar strategies to prevent or mitigate DRHL, NRHL and ARHL.

## Introduction

The most important paradigm recognized and highlighted in this article is that inflammation in any tissue, organ and system, behaves as a beneficial host reaction aimed at protecting individuals from infections and tissue injury. Moreover, inflammation can help to establish an immunological memory that the organism can use later to generate a better response to a particular infectious agent (Gilroy and De Maeyer, 2015; Headland and Norling, 2015). Therefore, rather than to prevent inflammation, any clinical strategy should be aimed at facilitating its rapid, safe and complete resolution.

**Figure 1:**
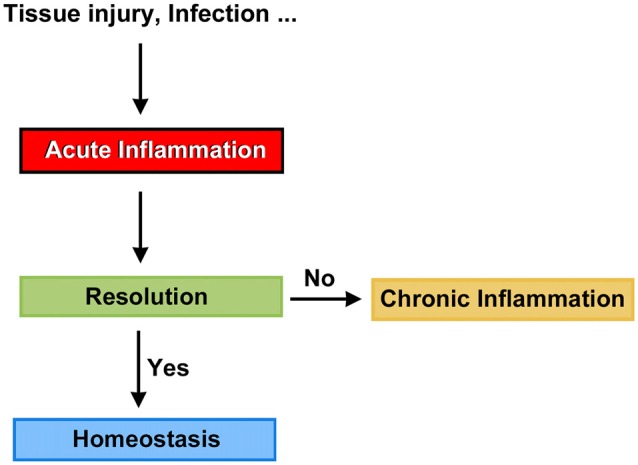
Potential fates for acute inflammation.

Mammals are able to detect the presence of pathogen agents and tissue injury, and initiate complex tissue repair and wound healing programs. At the front line of the host defense mechanism is acute inflammation, a short-term physiologic response aimed to return, at least in part, the organism to the normal phenotype. Not surprisingly, when the timely resolution of inflammation fails, it progresses to chronic inflammation, a condition that can persist for months and even years (Figure 1). Chronic inflammation is linked to the pathogenesis of a number of diseases such as atherosclerosis, type 2 diabetes, rheumatoid arthritis and Alzheimers (Medzhitov, 2008, 2010; Tabas and Glass, 2013), and likely act as a predisposing factor to carcinogenesis (Lee et al., 2013). Thus, the resolution of inflammation may be a crucial target for new therapeutic avenues, and we believe that clinical strategies seeking the timely resolution of inflammatory processes in the cochlea should be considered an important part of the conceptual framework needed to prevent auditory dysfunction.

## The Inflammatory Response and Its Resolution

### General Concepts

**Figure 2:**
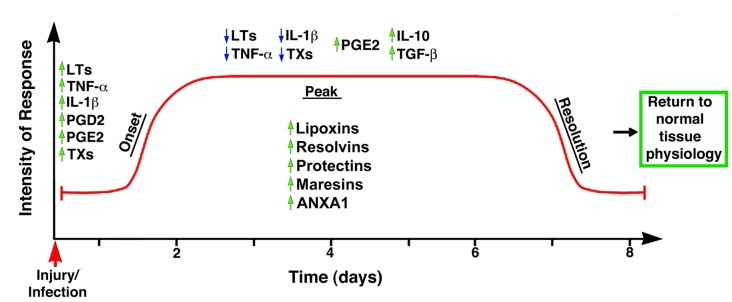
Temporal representation of the biochemical events associated with the onset and resolution of inflammation. The early phase of inflammation is characterized by the up-regulation (green arrows) of pro-inflammatory mediators such as leukotrienes (LTs), tumor necrosis factor-α (TNF-α), interleukin 1β (IL-1β), prostaglandin D2 (PGD2) and thromboxanes (TXs). Importantly, the anti-inflammatory mediator PGE2 is also up-regulated at this phase, indicating the controlled nature of inflammatory responses. The peak of the inflammatory response coincides with the start of the resolution phase, with down-regulation (blue arrows) of TXs, IL-1β, LTs and TNF-α, and up-regulation of anti-inflammatory cytokines such as PGE2, IL-10 and TGF-β; simultaneously, the synthesis and/or release of pro-resolution mediators (e.g., LX, resolvins, protectins, maresins, ANXA1) stop further infiltration of leukocytes and facilitate the removal of apoptotic cells, leading to the successful termination of the inflammatory response and the return to the tissue to its homeostatic condition. Modified from Maderna and Godson (2009).

Until recently, the common view on chronic inflammation was that it resulted from exaggerated pro-inflammatory signals during the acute phase, and that its resolution was a passive process mediated by metabolites of the same pro-inflammatory mediators. In other words, that chemo-attractants and other molecules associated with the inflammatory response would eventually dissipate, and the system would automatically reset to its initial stage (Robbins and Cotran, 1979; Tauber and Chernyak, 1991; Serhan, 2011). Studies in the last two decades, however, have led to the formulation of a new paradigm based on the premise that resolution of inflammation results from the engagement and activation of specific genetic, cellular and molecular programs (Perretti, 2015). Thus, it is currently accepted that acute inflammation is terminated by a biosynthetically active process, regulated by endogenous signaling pathways driven by specialized pro-resolving mediators and receptors that: (1) switch from the production of pro-inflammatory mediators to pro-resolution mediators; (2) turn off pro-inflammatory signaling pathways; (3) induce apoptosis of previously recruited inflammatory cells; (4) stimulate the clearance of apoptotic cells by phagocytes; and (5) reinstate, either partially or totally, homeostatic conditions (Alessandri et al., 2013). It has now become evident that the peak of the acute inflammatory response is the beginning of resolution (Serhan and Savill, 2005), with the simultaneous presence of pro-inflammatory and pro-resolution mediators in order to ensure safe cell death and removal, that is, preventing the activation of inflammatory and immune effectors (Gilroy et al., 2004; Hallett et al., 2008; Maderna and Godson, 2009; Perretti and D’Acquisto, 2009; Iqbal et al., 2011; Figure 2). This concept suggests a complex balance between pro-inflammatory and anti-inflammatory events taking place, at least partly, in parallel (Serhan and Savill, 2005; Sugimoto et al., 2016a). Moreover, it strongly corroborates that inflammation is programmed to stay within limits, both spatially and temporally, and to ultimately lead to an active process of completion (Perretti, 2015).

The current mainstay approach for treating inflammation-induced diseases is based on inhibiting the synthesis or activities of the pro-inflammatory mediators. Although there has been success with some of these anti-inflammatory therapies, there are considerable limitations. In particular, the advantages of anti-inflammatory drugs are usually decreased by three factors: redundancy, compensatory pathways and necessity (Tabas and Glass, 2013). For example, many molecules are at work in an inflammatory process, some of them with identical function (redundancy), and targeting one or a few of them may not be enough to obtain significant beneficial results. Likewise, inhibition of one pro-inflammatory pathway may just trigger a compensatory response involving an alternative pathway. Finally, inflammation is a protective reaction (necessity) and, even if the previous two challenges are successfully overcome, the risks associated with inhibiting a natural defense mechanism are often unacceptable (Tabas and Glass, 2013). Thus, there is an increasing awareness that pro-resolution-based strategies may have even more potential than anti-inflammatory therapies for the treatment of multiple diseases (Gilroy et al., 2004; Rossi et al., 2007; Hallett et al., 2008; Serhan et al., 2008; Duffin et al., 2010).

### Pro-Resolution Mediators and their Receptors

In recent years, interest for the resolution phase of inflammatory responses led to the discovery of several specific pro-resolving mediators of diverse nature, including lipids (Serhan et al., 2014), proteins and peptides (Perretti and Dalli, 2009), a purine (Köröskényi et al., 2011; Csóka et al., 2012; Haskó and Cronstein, 2013), gaseous mediators (Wallace et al., 2015), and neuromodulators (Pavlov and Tracey, 2012; Mirakaj et al., 2014). Specialized pro-resolving mediators not only work in inflammatory responses, but they also have important functions in host defense, pain, organ protection and tissue remodeling (Serhan et al., 2015a). More important, synthetic forms of these mediators have potent effects when administered *in vivo* (Serhan et al., 2014), increasing their clinical value.

**Figure 3:**
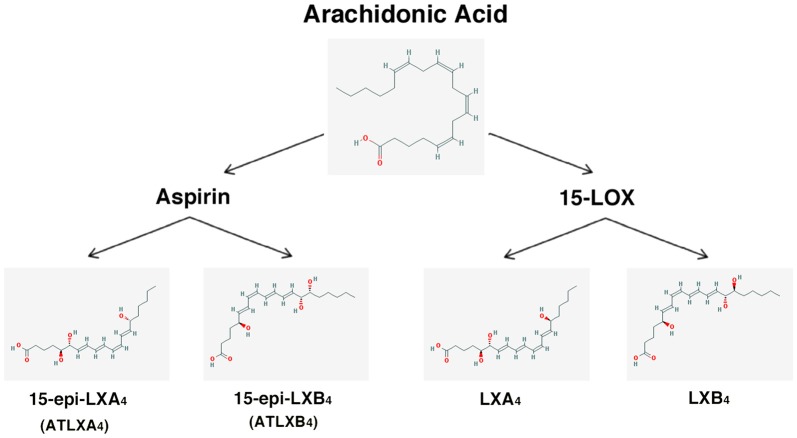
Scheme showing the formation of lipoxins and aspirin-triggered lipoxins from arachidonic acid. The 2-D structures were obtained from the PubChem Substance and Compound database with the following chemical structure identifiers (CID): 444899 (AA), 5280914 (LXA4), 5280915 (LXB4), 9841438 (ATLXA4), 9928453 (ATLXB4; National Center for Biotechnology Information, 2017).

**Figure 4:**
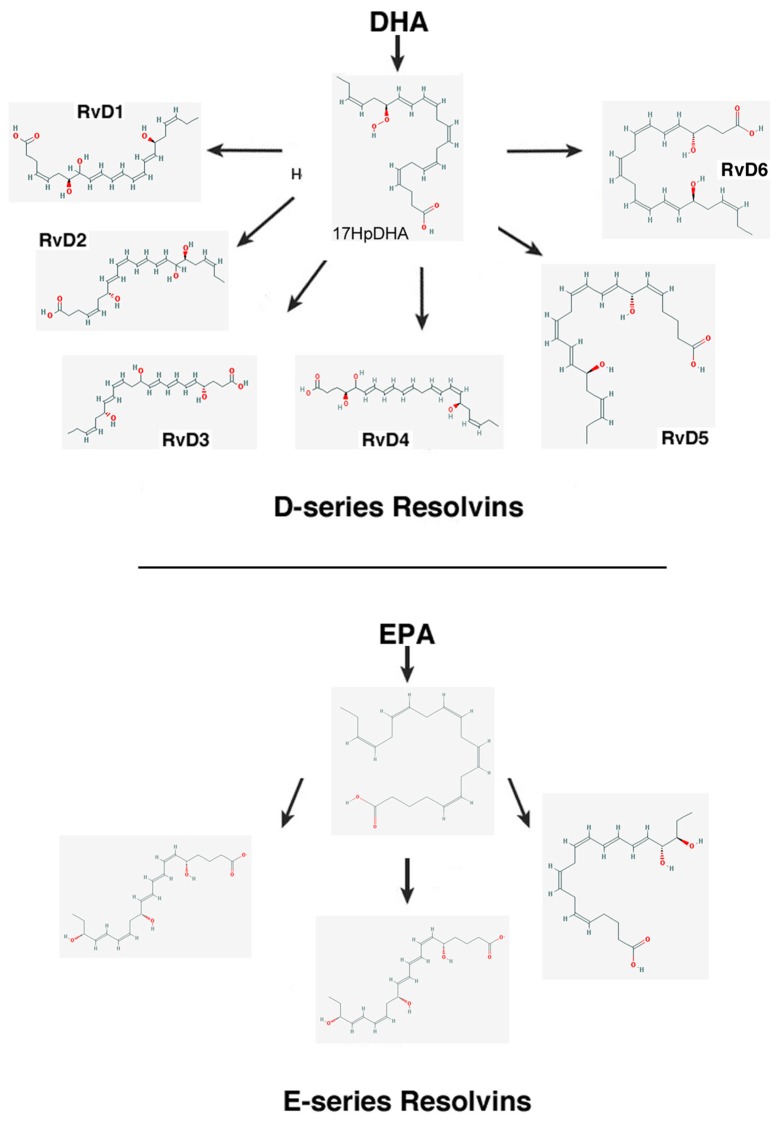
Scheme showing the biosynthesis of D-resolvins and E-resolvins from docosahexaenoic acid (DHA) and eicosapentaenoic acid (EPA), respectively. The 2-D structures were obtained from the PubChem Substance and Compound database with the following CID: 52921992 (17HpDHA), 16061135 (RvD1), 11383310 (RvD2), 53477497 (RvD3), 53477505 (RvD4), 16061139 (RvD5), 25073193 (RvD6), 446284 (18HpETE), 91820117 (RvE1), 16061125 (RvE2), 56848721 (RvE3; National Center for Biotechnology Information, 2017).

**Figure 5:**
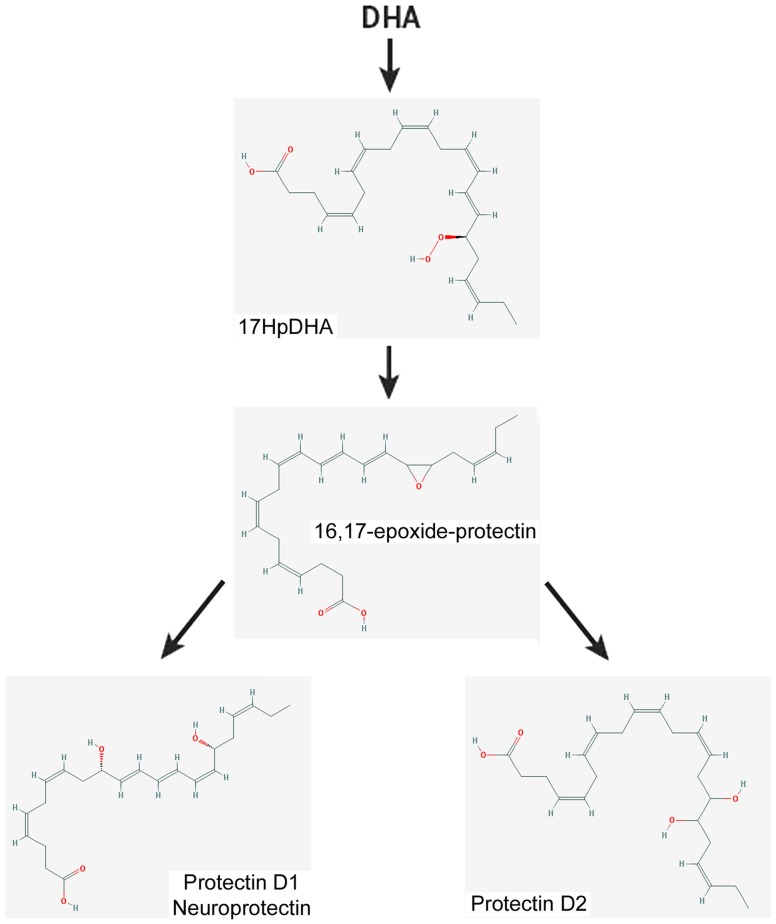
Scheme showing a simplified biosynthetic pathway for protectin 1 and protectin 2 from DHA. The 2-D structures were obtained from the PubChem Substance and Compound database with the following CID: 16061141 (16,17-epoxide-protectin), 16042541 (PRD1), 16061147 (PRD2; National Center for Biotechnology Information, 2017).

**Figure 6:**
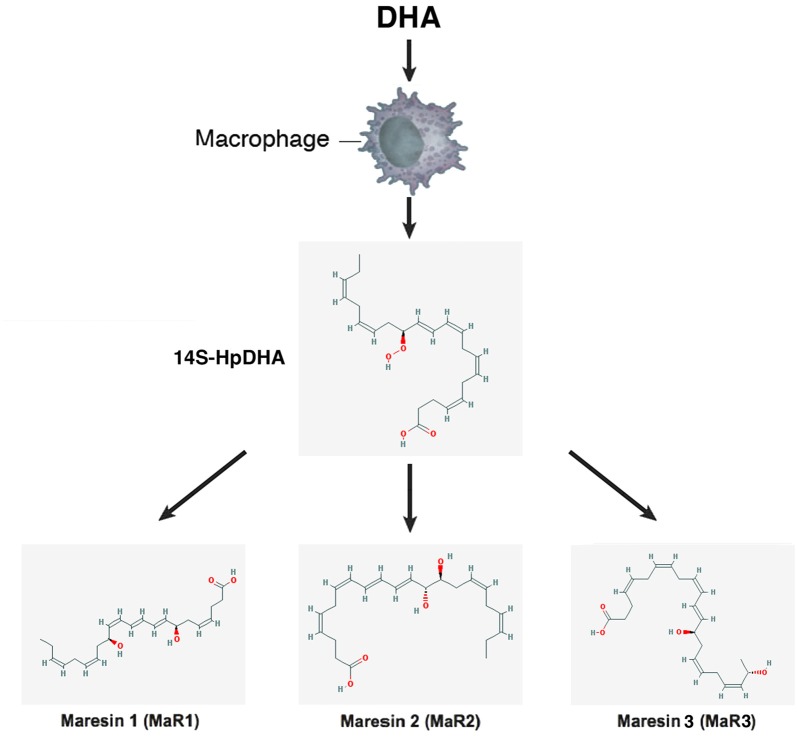
Scheme showing a simplified biosynthetic pathway for maresins. Macrophages convert DHA to the 13S-14S-epoxy-maresin intermediate, and from there MaR1, Mar2 and MaR3 are generated via soluble hydrolases. The 2-D structures were obtained from the PubChem Substance and Compound database with the following CID: 53477498 (14S-HpDHA), 60201795 (MaR1), 101894912 (MaR2), 52921996 (MaR3; National Center for Biotechnology Information, 2017).

**Figure 7:**
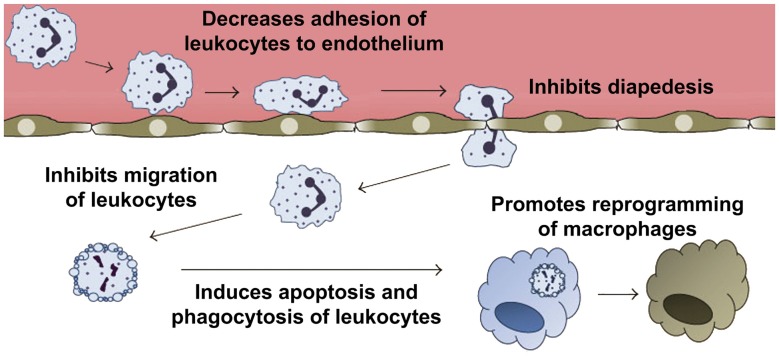
Anti-inflammatory and pro-resolving effects of annexin A1 (ANXA1). Pharmacological administration of ANXA1 or its mimetic N-terminal peptides results in decreased migration and adhesion of leukocytes to the endothelium and inhibition of their passage through the walls of the blood vessels (diapedesis). In addition, ANXA1 is able to induce apoptosis, and clearance of apoptotic leukocytes by macrophages. Furthermore, ANXA1 promotes the reprogramming of macrophages toward a pro-resolving phenotype, and even the generation of non-professional phagocytic cells. Modified from Sugimoto et al. (2016b).

To date, the most important pro-resolution mediators described in the literature are:

– *Lipoxins* (lipoxygenase interaction products; LX), eicosanoids generated *in vivo* from arachidonic acid (Serhan, 2005; Ryan and Godson, 2010; Figure 3). LX are involved in the inhibition of neutrophil and eosinophil recruitment and activation, while enhancing the recruitment of monocytes to sites of injury (Papayianni et al., 1995; Maddox et al., 1997; Wada et al., 2001). In addition, they are able to directly stimulate the expression of anti-inflammatory and pro-resolution genes (Qiu et al., 2001) as well as to regulate NF-kB activation (Decker et al., 2009). Further, they are known to stimulate the clearance of apoptotic cells by macrophages (Reville et al., 2006). Chronic inflammation has been associated with deficient LX biosynthesis, which makes tissues unable to resolve acute inflammatory reactions (Bandeira-Melo et al., 2000; Pouliot et al., 2000; Bonnans et al., 2002; Karp et al., 2004). Importantly, it has been shown that LX can stimulate the expression of ZO-1, claudin and occludin in cultured epithelial cells (Grumbach et al., 2009), suggesting that they could have a protective role in the maintenance of the tight-junction barrier at the reticular lamina of the organ of Corti.
Preeminent amongst lipoxins are the positional isomers LXA4 and LXB4 (Pettitt et al., 1991; Rowley et al., 1991; Serhan, 2005; Figure 3). Interestingly, aspirin-mediated acetylation of the COX-2 enzyme led to the generation of 15-epi-LX, known as aspirin-triggered LX (ATL; Serhan, 2005). Thus, part the beneficial effects of aspirin in humans may be associated with the endogenous biosynthesis of ATL mimicking the bioaction of native LX. While adequate stimulation induces the near immediate generation of LX and ATL, they are also rapidly inactivated by dehydrogenation and reduction to form triene-containing compounds (e.g., LXA4 into the biologically inactive compounds 15-oxo-LXA4, 13,14-dihydro-15-oxo-LXA4 and 13,14-dihydro-LXA4) by metabolic enzymes present in leukocytes of the monocyte/macrophage lineage, mainly monocyte/MΦ (Serhan et al., 1993; Maddox and Serhan, 1996; Clish et al., 2000; Romano, 2006). Although ATL are also converted to their biologically inactive 15-oxo-metabolites, the process is slower, suggesting that they possess an extended activity *in vivo* (Serhan et al., 1995). As a result of the short lives of the endogenous mediators, stable analogs for both LX and ATL were developed that can resist metabolism, maintaining their structural integrity (Chiang et al., 2005). Studies in animal models suggest that LX analogs could also be useful in humans, providing the rationale for development of innovative anti-inflammatory drugs (Romano, 2005) and “resolution-targeted” therapies (Chiang et al., 2005).
LX are known to bind the G-protein coupled receptor ALX/FRP2 (Chiang et al., 2006). This receptor has been detected on human neutrophils, eosinophils, airway epithelium, monocytes, macrophages, T cells, synovial fibroblasts and intestinal epithelial cells (Fiore et al., 1994; Maddox et al., 1997; Bonnans et al., 2006; Chiang et al., 2006; Barnig et al., 2013). Importantly, these receptors have also been localized in guinea pig cochlear cells (Kalinec et al., 2009). ALX/FRP2 expression is regulated by inflammatory mediators, transcription factors and epigenetic mechanisms, and LXA4 is known to increase ALX/FRP2 expression by activating its promoter in a positive-feedback fashion (Simiele et al., 2012).
– *Resolvins* (resolution phase interaction products) are ω-3 essential fatty acids derivatives with powerful multilevel anti-inflammatory and pro-resolving properties (Serhan et al., 1984, 2000a; Pettitt and Rowley, 1991; Arita et al., 2005). They are termed D-series resolvins (RvD) if generated from docosahexaenoic acid (DHA; 22:6ω-3), or E-series resolvins (RvE), if the biosynthesis is initiated from eicosapentaenoic acid (EPA; 20:5ω-3; Serhan et al., 2000a; Serhan and Chiang, 2008; Figure 4). Aspirin is known to oxidize DHA (Kusunoki et al., 1992; Serhan et al., 2004a,b), generating aspirin-triggered D-resolvins (ATR-D; Kusunoki et al., 1992; Serhan et al., 2000b; Serhan and Chiang, 2004). A novel resolvin subfamily, generated from docosapentaenoic acid (DPA; 22:5ω-3) and termed T-series (RvT), has also been recently described (Dalli et al., 2015a).
Resolvins have a key role in the resolution of inflammatory responses, regulating the migration of neutrophils and resolution macrophages to sites of inflammation as well as reducing the levels of pro-inflammatory mediators (Serhan et al., 2015a). RvDs and RvEs also promote phagocytosis of apoptotic neutrophils (Schwab et al., 2007; Krishnamoorthy et al., 2010), reduce activation and aggregation of platelets (Dona et al., 2008), and regulate the function of T and B cells (Ariel et al., 2006; Ramon et al., 2012). The just discovered RvTs, on the other hand, could be crucial in the resolution of inflammation triggered by bacterial infection (Dalli et al., 2015a). In mice, the local application of RvD1 significantly decreases the number of apoptotic cells and macrophages in diabetic wounds, accelerating wound closure and granulation tissue formation (Spite et al., 2014). In animal models of sterile inflammation, RvE1 decreases the expression of the genes encoding TNF-α, IL-1β and VEGF (Jin et al., 2009) as well as IL-12 production (Poorani et al., 2016). RvEs and RvDs are more active than their precursors EPA and DHA, and they are known to induce significant effects even at nanomolar concentrations. They have potent *in vivo* actions in many important human pathologies, such as obesity and diseases affecting the vascular (Miyahara et al., 2013), airway (Levy and Serhan, 2014), and ocular systems, as well as in reactions involving pain, fibrosis and wound healing (Serhan and Chiang, 2013; Spite et al., 2014).
RvD1 and its aspirin-triggered epimer RvD1 (ATR-D1) bind the ALX/FRP2 receptor, just like LX (Fiore et al., 1994; Chiang et al., 2006; Krishnamoorthy et al., 2012). Interestingly, RvD1 and ATR-D1, as well as RvD3, and RvD5, have also been shown to bind and signal through a specific receptor termed RvD1-R (Sun et al., 2007; Krishnamoorthy et al., 2010, 2012; Chiang et al., 2012; Dalli et al., 2013b). It appears that RvD1 differentially interacts with RvD1-R during periods of homeostasis and with ALX/FRP2 during the resolution phase of inflammation (Krishnamoorthy et al., 2012). In turn, RvD2 and RvE have specific receptors termed, respectively, RvD2-R (Chiang et al., 2015) and RvE-R (Arita et al., 2005; Campbell et al., 2007; Parolini et al., 2007; Cash et al., 2008; Du and Leung, 2009; Barnig et al., 2013).
– *Protectins* also derive from DHA (Hong et al., 2003; Serhan et al., 2006; Figure 5). Protectins, like resolvins, control the magnitude and duration of inflammation in animal models (Schwab et al., 2007; Serhan and Petasis, 2011), fight bacterial and viral infections (Chiang et al., 2012), and can increase animal survival (Serhan and Chiang, 2013).
Two members of the family have been described, protectin-D1 and protectin-D2 (Dalli et al., 2013a). PD1, originally identified in neural tissues (murine brain cells and human microglial cells), is also known as neuroprotectin (Kohli and Levy, 2009). The identity of the protectin receptor/s is not yet known, although it has been proposed that, in humans, they bind to high affinity sites in the plasma membrane of neutrophils (Marcheselli et al., 2010). Interestingly protectins have been shown to regulate the expression of the Peroxisome Proliferator-Activated Receptor-γ (PPAR-γ; White et al., 2015), a member of a nuclear hormone receptor superfamily that, acting as transcription factors, regulate inflammation, immune responses and metabolic processes that influence lipid metabolism, glucose homeostasis, cell differentiation, obesity and cancer (Chinetti et al., 2003; Moraes et al., 2006).
– *Maresins* (macrophage mediators in resolving inflammation) are anti-inflammatory and pro-resolving lipid mediators generated by macrophages from DHA (Serhan et al., 2009; Figure 6). To date, three members of the family have been described, MaR1, MaR2 and MaR3 (Dalli et al., 2013a), which display potent anti-inflammatory and pro-resolving actions even at the nanogram range (Poorani et al., 2016). Maresins are known to stimulate phagocytosis of polymorphonuclear (PMN) by macrophages (Serhan et al., 2015b), increase the number of regulatory T cells and decrease the production of interleukins 5 and 13 (IL-5, IL-13; Krishnamoorthy et al., 2015), as well as inhibit the production of leukotriene B4 (LTB4; Serhan et al., 2015b), contributing to the completion of the resolution phase of inflammatory responses.
The identity of the receptor/s through which maresins signal is still unknown.
**NOTE:** Several sulfido-conjugates of maresins, protectins and D-resolvins are also biologically active (Dalli et al., 2014, 2015b). These sulfido-conjugates are usually produced from DHA by activated phagocytes, and they are very effective in stimulating inflammatory resolution and tissue regeneration (Duvall and Levy, 2016).
– *Annexin A1* (ANXA1) is a powerful anti-inflammatory and pro-resolving protein. ANXA1 synthesis and release is regulated by glucocorticoids (Sugimoto et al., 2016b), and it is considered a pivotal homeostatic mediator (Perretti and Dalli, 2009), and an important modulator of both the innate and adaptive immune systems (D’Acquisto et al., 2008; Perretti and D’Acquisto, 2009). Experimental evidence suggests that ANXA1, in addition to decreasing pro-inflammatory cytokines while increasing the production of immunosuppressive and pro-resolving molecules, is able to induce macrophage reprogramming toward a resolving phenotype, and even converting somatic cells into non-professional macrophages (Sugimoto et al., 2016a; Figure 7). Several studies indicate that the anti-inflammatory and pro-resolution activity of ANXA1 is associated with its N-terminus. Importantly, short synthetic peptides from this domain retain the receptor binding specificity of the full protein and have most of their effects, but they are more resistant to inactivation (Perretti and D’Acquisto, 2009).
Since many of the cellular and molecular processes associated with the anti-inflammatory properties of glucocorticoids are, actually, modulated by ANXA1, pharmacologic interventions based on ANXA1 could be equally effective as steroids without their negative side effects. The association of ANXA1 with lipid droplets in auditory Hensen cells from guinea pigs, as well as its potential role in the resolution of cochlear inflammation, have been discussed in a recent review (Urrutia and Kalinec, 2015).
ANXA1, as well as short synthetic peptides from its N-terminal domain, specifically bind to ALX/FPR2, the same receptor that binds LXA4 and resolvins D1/E1(Perretti and D’Acquisto, 2009). Thus, the ALX/FPR2 receptor is shared by a variety of peptide/protein and lipid ligands, mediating many functions of relevance for inflammation (Anong et al., 2009). The promiscuity of ALX/FPR2 seems to be linked to a network of both pro-inflammatory and pro-resolving signaling pathways (Le et al., 2007; Brancaleone et al., 2011). It was demonstrated that distinct ALX/FPR2 domains are required for signaling by different agonists; for instance, while ANXA1-mediated signaling involves the N-terminal region and extracellular loop II of ALX/FPR2, LXA4 activates ALX/FPR2 by interacting with the extracellular loop III and its associated transmembrane domain (Bena et al., 2012).
– *Galectins*, a family of glycan-binding proteins, are currently considered key players in several programs that control maturation, activation, differentiation, polarization, trafficking, cytokine synthesis and viability of immune cell populations (Rabinovich and Toscano, 2009; Mendez-Huergo et al., 2014; Rabinovich and Conejo-García, 2016). By crosslinking specific glycoconjugates, different members of the galectin family (15 members identified to date) behave as either pro-inflammatory or anti-inflammatory agents, regulating the initiation, amplification and resolution of acute and chronic inflammatory responses (Rabinovich et al., 2002; Rubinstein et al., 2004). Several studies identified Gal-1, Gal-3 and Gal-9 as direct players in the modulation of acute and chronic inflammatory diseases, autoimmunity and cancer, and are increasingly used as targets for drug discovery (Norling et al., 2009). Gal-1 has been associated with a range of anti-inflammatory effects on various cells types (Rabinovich et al., 2000; Dias-Baruffi et al., 2003; La et al., 2003). Gal-3, in turn, is widely pro-inflammatory, and it appears to be involved in the transition to chronic inflammation (Henderson and Sethi, 2009). Gal-3 is known to enhance the phagocytic capabilities of neutrophils, a fact that may in part account for its protective role in infections (Farnworth et al., 2008). Finally, the ability of Gal-9 to induce T-cell apoptosis makes it a potent anti-inflammatory protein (Tsuchiyama et al., 2000; Zhu et al., 2005; Katoh et al., 2007), with several pro-resolution properties including the increase of leukocyte apoptosis and phagocytic clearance (Iqbal et al., 2011).
After being secreted galectins bind to glycoprotein targets, forming galectin–glycan complexes that regulate the organization of glycosylated receptors as well as their internalization and signaling (Rabinovich and Croci, 2012; Thiemann and Baum, 2016). Thus, they are able to stimulate different signaling cascades associated with inflammation (Norling et al., 2009; Rabinovich and Toscano, 2009; Blidner et al., 2015) and regulate the activity of immune cells by controlling the function of relevant glycosylated receptors.
– *Adenosine* is a ubiquitous metabolite of ATP generated as a result of cellular injury or stress, and is released from cells via specific transporters or during apoptosis or necrosis (Haskó and Cronstein, 2013). Adenosine is an important immunosuppressive and tissue-healing factor, and its production and extracellular concentrations are significantly increased in inflammation (see Aherne et al., 2011 and references therein). In the auditory system adenosine has been associated with protective mechanisms against noise- and drug-related hearing loss (for a review, see Vlajkovic et al., 2009). In mice, adenosine release is known to be induced by aspirin (Cronstein et al., 1999), and it is possible that adenosine could be the real effector of some of the pro-resolution properties of aspirin.
Adenosine interacts with purinergic receptors type 1 (P1) on the plasma membrane of inflammatory and immune cells (Bours et al., 2006), regulating their function and limiting inflammatory tissue destruction (Haskó and Cronstein, 2004). Adenosine-activated P1 receptors are further divided into A1, A2 and A3 subtypes, with A1 and A3 inhibiting and A2 stimulating adenyl cyclase after being activated by adenosine; in addition, the A2 subtype is subdivided into A2A (high-affinity) and A2B (low-affinity) adenosine receptors (Ralevic and Burnstock, 1998). Since the particular effects of adenosine depend on which receptor is activated, the physiological effects of adenosine will depend on the relative expression of their receptors in a particular cell, tissue, or organ (Ralevic and Burnstock, 1998). In mammalian cochleae, adenosine receptors A1 and A2A are abundantly expressed in inner hair cells (IHCs), Deiters cells and spiral ganglion neurons, whereas A3 is localized in IHCs and outer hair cells (OHCs), as well as in Deiters, pillar, Hensen, Claudius, spiral ganglion, inner and outer sulcus cells (Vlajkovic et al., 2009).
– *NO, H_2_S and CO*, nitric oxide, hydrogen sulfide and carbon monoxide, respectively, are gaseous substances that can act as signaling molecules (Wallace et al., 2015). NO has the ability to regulate apoptosis of inflammatory cells, with lower concentrations of NO usually being cytoprotective, while supra-physiological concentrations trigger cell death (Kim et al., 1999; Brüne, 2005). These pro- and anti-apoptotic properties are cell-specific, and depend largely on the NO isoforms involved (Taylor et al., 2003). In inflammatory cells, low concentrations of endothelial and neuronal isoforms of NO synthase (eNOS and nNOS) have a protective effect, whereas higher concentrations from the inducible isoform (iNOS) are more likely to induce apoptosis (Nicotera et al., 1997). Since the resolution of inflammatory processes requires death and clearance of inflammatory cells, NO-mediated regulation of apoptosis may be critical for ensuring the return to homeostasis.
H_2_S exerts potent inhibitory effects on a wide range of leukocyte functions (Zanardo et al., 2006; Pálinkás et al., 2015). Importantly, H_2_S is an avid scavenger of other cytotoxic substances, including peroxynitrite (Whiteman et al., 2004), superoxide anion (Muzaffar et al., 2008), hypochlorous acid (Whiteman et al., 2005) and hydrogen peroxide (Whiteman et al., 2010), all of them important for oxidative stress. In addition, ANXA1 mediates some of the anti-inflammatory actions of H_2_S (Brancaleone et al., 2014), and there is strong evidence that H_2_S helps to restore tissue function by up-regulating enzymes that drive tissue repair and preserve mitochondrial function (Goubern et al., 2007; Lagoutte et al., 2010; Mimoun et al., 2012).
CO is produced via the inducible isoform of the enzyme heme oxygenase (HO-1), which is a sensor of cellular stress, and it has been shown that the CO it generates may limit tissue injury (Motterlini and Foresti, 2014). Like NO and H_2_S, CO has anti-apoptotic, anti-inflammatory and anti-proliferative effects, and these functions seem to be associated, at least in part, with their influence on oxidative stress, redox signaling and cellular respiration (Motterlini and Foresti, 2014). In the clinic, in addition to the oral administration of CO-releasing molecules (“CO-RMs” or “CORMs”), it is relatively common to use CO as an inhaled gas (Wallace et al., 2015). It has been reported that inhaled CO reduces neutrophil infiltration and stimulates the activity of HO-1 and phagocytosis by resolution macrophages (Chiang et al., 2013). Importantly, inhaled CO also increases the production of RvD1 and Mar1 while decreasing LTB4 (Chiang et al., 2013).
The gaseous mediators NO, H_2_S and CO, in contrast to other signaling molecules, do not have specific receptors. Their effects result from direct interaction with a great number of different proteins and genes (Wallace et al., 2015).
– *Neuromodulators*, like acetylcholine and netrin-1, control immune function and anti-inflammatory responses via a vagus nerve-mediated reflex (Pavlov and Tracey, 2012). Acetylcholine receptors, including α7nAChR, act as molecular targets for the vagus-mediated signals, and several α7nAChR agonists have shown anti-inflammatory properties in human volunteers (Kitagawa et al., 2003). The local expression of netrin-1, an axonal guidance molecule known to stimulate the production of resolvins, is also regulated by the vagus nerve (Ly et al., 2005; Mirakaj et al., 2010, 2011; Aherne et al., 2012). In addition to promoting the generation of resolvins, Netrin-1 activates resolution by decreasing the recruitment of PMN cells *in vitro* and *in vivo* while increasing the recruitment of monocytes and its uptake of apoptotic PMNs (Mirakaj et al., 2014). Interestingly, netrin-1 was localized in the early postnatal rat and mouse cochlea, and it has been suggested that it could have an important role in promoting the growth of spiral ganglion cells’ neurites as well as guiding their axons (Gillespie et al., 2005; Lee and Warchol, 2008). Moreover, it has been shown that insulin-like growth factor 1 (IGF-1) up-regulates the expression of netrin-1 in the neonatal mouse inner ear (Hayashi et al., 2014), and recent results suggest that netrin-1 could be a key mediator of the protective role of IGF-1 against the ototoxic effects of aminoglycosides (Yamahara et al., 2017).
– Aspirin as a pro-resolution mediator
Aspirin is a potent inhibitor of cyclo-oxygenases (COX) and lipoxygenases (LOX), interfering with the synthesis of pro-inflammatory mediators (Forge and Schacht, 2000; Gilroy, 2005b). However, unlike many other anti-inflammatory agents considered resolution-toxic because they delay complete resolution (Gilroy et al., 1999; Schwab et al., 2007), aspirin promotes resolution mechanisms (Gilroy and Perretti, 2005; Serhan, 2007). At high doses (~1 g), aspirin is anti-inflammatory, but it is pro-resolution at lower doses (~81 mg) because of the synthesis of the pro-resolution mediator ATLA4 and up-regulation of its receptor ALX/FRP2. Since ATLA4 inhibits the pro-thrombotic eicosanoid thromboxane, a low dose aspirin is commonly used for the prevention of vascular diseases (Morris et al., 2009).
In addition to these effects, and as already mentioned, aspirin-acetylated COX-2 is the origin of ATL and ATR (Serhan, 2005). Thus, it is argued that the most important mechanism of action of aspirin is the induction of pro-resolution mediators (Gilroy and Perretti, 2005; Gilroy, 2005a,b). Importantly, aspirin may acetylate COX-2 in one cell type and ATL and ATR be generated in a different one in a process known as transcellular metabolism (Serhan et al., 2000a; Gilroy et al., 2004; Gonzalez-Períz and Claria, 2007). For example, 15-hydroxyeicosatetraenoic acid (15-HETE), generated by aspirin acetylation of COX-2 in endothelial cells, may be released and then metabolized to ATL by inflammatory cells. These relatively unrecognized pathways and compounds may represent new ways to develop novel “resolution-targeted” therapeutics (Chiang et al., 2005).
There is no receptor for aspirin, but it is known to stimulate a variety of receptor-mediated signaling pathways, for example, by the production of ATL and ATR (Gilroy, 2005b). The main receptor for ATL and ATR-D1 is ALX/FRP2, the same molecule that binds LX, ANXA1 and RvD1/E1 (Fiore et al., 1994; Chiang et al., 2006; Krishnamoorthy et al., 2012). Moreover, ATR-D1 also activates RvD1-R, the specific receptor known to bind RvD1, RvD3 and RvD5 (Sun et al., 2007; Krishnamoorthy et al., 2010, 2012; Chiang et al., 2012; Dalli et al., 2013b). Thus, indirectly through the activation of the signaling pathways mediated by these receptors (as well as others mechanisms such as the release of adenosine), aspirin has a unique ability to induce a variety of pro-resolution effects.
There is considerable interest in elucidating whether aspirin also favors resolution in humans (Morris et al., 2009, 2010). It is already known that, in humans, low-dose aspirin triggers ATL production just like in animal models (Chiang et al., 2004). Thus, the combination of aspirin with ω-3 essential fatty acids might have a beneficial impact on diseases associated with inflammation in many organs, including the cochlea. Moreover, it has been suggested that aspirin could slow down the progression of age-related hearing loss (ARHL; Lowthian et al., 2016).

### Resolution of Inflammation in the Mammalian Cochlea—General Concepts

**Figure 8:**
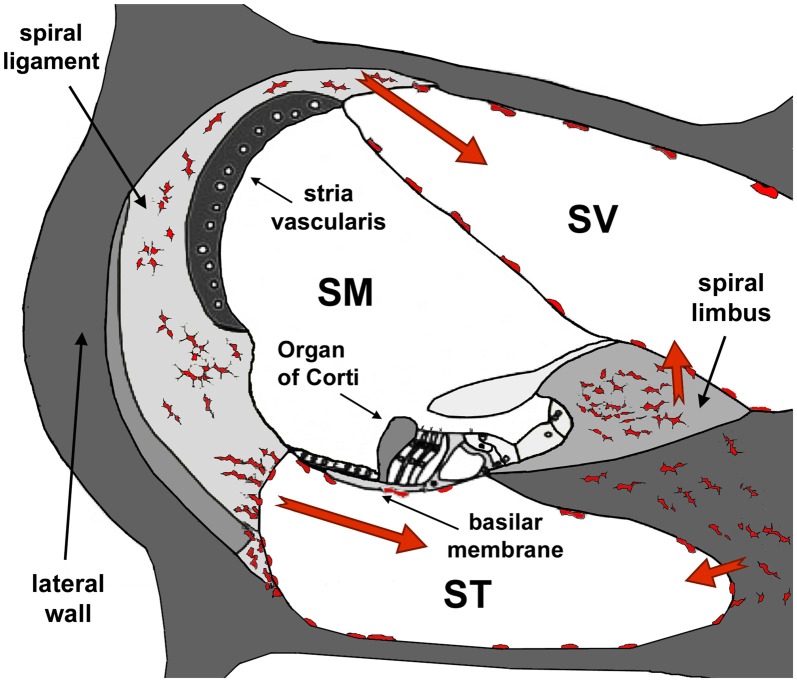
Diagram of a section of the cochlea. Note the three cochlear ducts, the scala vestibule (SV), the scala media (SM) and the scala tympani (ST). The auditory organ, the organ of Corti, sits at the SM. At the onset of inflammation, leukocytes and resident macrophages in the lateral wall and the spiral limbus migrate to the SV and the ST but, except in cases of extreme cochlear damage, they do not penetrate into the SM. Modified from Urrutia and Kalinec (2015).

For many years, the cochlea was considered an “immune-privileged” organ because of the presence of a tight junction-based blood-labyrinth barrier (BLB; Harris, 1983, 1984; McCabe, 1989). A number of more recent studies, however, showed that resident macrophages are always present in the cochlear lateral wall as well as in the spiral limbus and the scala tympani (ST) side of the basilar membrane (Frye et al., 2017), and they are activated by various types of insults, including noise exposure, ischemia, mitochondrial damage and surgical stress (Hirose et al., 2005; Zhang W. et al., 2012; Fujioka et al., 2014; Figure 8). Moreover, experimental data suggests that BLB permeability is regulated by inflammatory cytokines released by macrophages in the spiral ligament and macrophage-like melanocytes in the stria vascularis (Zhang W. et al., 2012; Fujioka et al., 2014), and that inflammation would increase BLB permeability to some ototoxic drugs (Koo et al., 2015).

The association of inflammation with ototoxicity was originally based on evidence that glucocorticoids protected against sensorineural hearing loss (Kanzaki and Ouchi, 1981). Later studies demonstrated that the cochlea can mount inflammatory responses not only in response to pathogens but also to toxic insults mediated by drugs, noise or immune challenges (sterile inflammation; Rock et al., 2010). For example, several ototoxic drugs are known to induce cell apoptosis and inflammation in the cochlea both directly or through the generation of reactive oxygen species (ROS; Kaur et al., 2011; Oh et al., 2011). Noise trauma also induces an inflammatory response in the inner ear (Fujioka et al., 2006), and studies in mice suggest that chronic environmental noise exposure could induce cochlear damage and hearing loss via inflammatory processes (Tan et al., 2016). Moreover, it has been suggested that low-grade inflammation may be also linked to some of the auditory problems usually associated with aging (Lowthian et al., 2016), otitis media, meningitis and autoimmune inner ear disease (Gloddek et al., 1999; Trinidad et al., 2005; Caye-Thomasen et al., 2009). Furthermore, cochlear inflammation is a common result of cochlear surgery and the insertion of cochlear implants (Backhouse et al., 2008; Okano et al., 2008).

Akin to pathogen-induced inflammation, the resolution phase in sterile inflammation is also initiated by apoptosis and clearance of damaged cells (Medzhitov, 2008). The similar response is due to the fact that pathogens and the byproducts of cellular damage, known as damage-associated molecular patterns (DAMPs), stimulate the same pattern recognition receptors (PRRs; Kono and Rock, 2008; Zitvogel et al., 2010). Early inflammation caused by DAMP-PRR signaling is considered an evolutionarily preserved mechanism for controlling the spread of pathogens or necrotic tissue (Wood and Zuo, 2017). Interestingly, until recently the accepted paradigm was that apoptosis, the physiological form of cell death, occurred without DAMPs; necrosis, in contrast, was thought to lead to the generation of DAMPs, followed by activation of inflammatory and immune pathways (Kono and Rock, 2008). However, the idea that accidental necrosis would always elicit inflammation and immune responses, and that apoptosis would be anti-inflammatory, is a misconception. In some cases apoptotic cells trigger immune responses (Green et al., 2009), whereas cell necrosis can be executed in a regulated and safe manner (Garg et al., 2010).

Thus, in the cochlea, PRR activation rapidly leads to the activation of resident macrophages, the release of pro-inflammatory cytokines, and ROS production, causing apoptosis of damaged cells and infiltration of immune cells into the scala vestibule (SV) and the ST. The nature of the immune cells infiltrating the cochlea has been discussed in a recent review (Wood and Zuo, 2017), and will not be addressed here. The infiltrating cells transform into activated macrophages and express pro-inflammatory proteins (Yang et al., 2015). Whereas leukocytes are essential elements of the immune system, providing the first line of defense against invading pathogens, they require appropriate regulation to avoid tissue damage (Hallett et al., 2008; Headland and Norling, 2015). This is particularly important in the cochlea, where migrant leukocytes may disrupt the tight junction barrier at the reticular lamina in the organ of Corti. Without tight-junctions, the endolymph of the scala media (SM) would mix with the perilymph of the ST, eliminating the differences in electrical potential between these two chambers, shutting off the cochlear amplification mechanism and inducing apoptosis of outer hair cells (Kalinec et al., 2009). Consistent with this idea, leukocytes and macrophages are never found in the SM except in cases of extreme, irreversible cochlear damage (Hirose and Liberman, 2003; Hirose et al., 2005; Tornabene et al., 2006). Therefore, inflammatory responses in the cochlea must also be aimed at suppressing leukocyte migration and activation as well as promoting the clearance of apoptotic cells in the organ of Corti by non-professional phagocytes.

To the best of our knowledge, there is only one work exploring the presence of resolution mediators and receptors in the cochlea (Kalinec et al., 2009). Looking at the inner ear of guinea pigs, ANXA1 was localized in several cell populations lining the SM, particularly in Hensen cells of the organ of Corti (Kalinec et al., 2009). The majority of ANXA1 within cochlear Hensen cells was found stored inside lipid droplets, and experimental evidence suggests that it is released to the external milieu by a glucocorticoid-activated mechanism (Kalinec et al., 2009). ALX/FPR2, the receptor for ANXA1, LX A4/B4, RvD1/E1 as well as ATL and ATR, was also found expressed in the SM and cells lining the ST and the SV of the guinea pig cochlea, being particularly abundant in sensory IHCs and OHCs, Deiters and Pillar cells (Kalinec et al., 2009). It was speculated that ANXA1 released by Hensen cells could target these receptors to induce pro-resolution effects. Importantly, although low concentrations of LXA4 were detected, no evidence was found of glucocorticoid-induced release of LXA4 from any organ of Corti cells (Kalinec et al., 2009).

The absence of professional phagocytic cells during inflammatory responses in the SM and the organ of Corti supports the idea that supporting cells, working as non-professional phagocytes, would be responsible for clearing apoptotic hair cells (Abrashkin et al., 2006). However, the signals that mediate the clearance of dead organ of Corti cells are still unknown. Interestingly, ANXA1 has been implicated in promoting phagocytosis in two ways: by acting as an “eat me” signal on apoptotic cells (Arur et al., 2003) and as a receptor on the surface of professional and non-professional phagocytic cells to recognize exposed phosphatidylserine (PS) on cells undergoing apoptosis (Fan et al., 2004). It has been suggested that ANXA1 molecules might act as bridging proteins, linking apoptotic cells to neighbor cells, promoting the transformation of these adjacent cells into non-professional phagocytes, and then inducing the phagocytosis of the apoptotic cells (Fan et al., 2004). Thus, the massive release of ANXA1 from Hensen cells induced by glucocorticoids could be important for both stopping leukocyte migration into the SM and for facilitating the clearance of apoptotic hair cells by inducing the transformation of supporting cells in the organ of Corti to non-professional macrophages (Kalinec et al., 2009).

### Resolution of Inflammation and Drug-Related Hearing Loss

Drug ototoxicity, defined as a temporary or permanent inner ear dysfunction after drug exposure, is one of the most preventable causes of deafness (Yorgason et al., 2011). While several classes of drugs are ototoxic, platinum-based chemotherapy agents (e.g., cisplatin) and aminoglycoside antibiotics (e.g., gentamicin, streptomycin) are known to induce irreversible hearing loss; others like macrolide antibiotics, antimalarial medications, loop diuretics and some NSAIDs are known to cause reversible inner ear toxicity (Yorgason et al., 2006). Although their ototoxicity is well known, these drugs are frequently used in the clinic because in many cases their benefits outweigh their negative side effects.

Here, we will only review cisplatin and aminoglycoside antibiotics since, as central components of many pharmacotherapies, they are arguably the most clinically relevant ototoxic drugs.

#### Cisplatin

Cisplatin is a potent chemotherapeutic agent used in the treatment of a variety of cancers. Its administration, however, is commonly associated with severe nephrotoxicity, peripheral neuropathy and ototoxicity (Coradini et al., 2007). The association of inflammation with cisplatin treatment has been suggested mostly by the beneficial effect of glucocorticoids on cisplatin ototoxicity (Murphy and Daniel, 2011; Parham, 2011), and the reduction of cisplatin nephrotoxicity by the pro-resolution mediators aspirin and adenosine (Okusa, 2002; Ramesh and Reeves, 2004).

In the inner ear the toxic effects of cisplatin are characterized by progressive, bilateral and irreversible hearing loss, preferentially affecting high frequencies and characterized essentially by damage to the cochlea (Nakai et al., 1982). The primary site of cochlear toxicity is the OHC, but IHCs, spiral ganglion neurons and stria vascularis cells are also affected. At the cellular level, cisplatin induces a complex network of events, including generation of ROS and activation of inflammatory cytokines and stress signaling pathways (Boulikas and Vougiouka, 2003; Rybak et al., 2007). These events eventually lead to cell death, mostly via induction of apoptosis (Boulikas and Vougiouka, 2003).

Currently, oxidative stress –not inflammation—is considered the major cause of cisplatin-induced hearing loss. However, the use of anti-oxidants as a single clinical strategy for cisplatin-induced hearing loss is risky and many times ineffective, most likely because of the multiple physiological roles of ROS (discussed below, see “Anti-Oxidants” Section). On the other hand, cisplatin is able to induce endoplasmic reticulum stress in association with the unfolded-protein response (UPR; Mandic et al., 2003; Liu and Baliga, 2005; Yu et al., 2008), and UPR-triggered inflammation is now thought to be fundamental in the pathogenesis of several diseases (Zhang and Kaufman, 2008). Cisplatin is also known to induce apoptosis in proliferating cells by damaging the DNA through the formation of adducts (between different strands) and cross-links (in the same strand). However, DNA damage could be less critical in cochlear hair cells, since they do not proliferate.

Using a proteomic approach in HeLa cells, it was demonstrated that cisplatin is able to change the expression of nuclear proteins as well as to induce alternative splicing (Wu et al., 2010; Zhang G. et al., 2012). Importantly, one of the proteins identified as changing its expression pattern was the pro-resolution mediator ANXA1, which was found to significantly increase its expression after cisplatin exposure. Moreover, it was shown that ANXA1 knockdown significantly increased cisplatin-induced DNA damage (Zhang G. et al., 2012), a response consistent with the up-regulation of ANXA1 in the cisplatin-resistant cell line CNE2-CDDP (Chow et al., 2009). These results suggest that ANXA1-based pharmacological strategies could protect against cisplatin-induced cell damage.

#### Aminoglycoside Antibiotics

Aminoglycosides are one of the most frequently employed antibiotics in the clinic. In addition to their potent bactericidal activities, aminoglycosides possess less bacterial resistance, more post-antibiotic effects and, perhaps most important, they are inexpensive. However, they have serious side effects, including nephrotoxicity and irreversible hearing loss (Forge and Schacht, 2000). Although these drugs are most frequently used in Third-World countries, where they usually are the only economically affordable antibiotics, their toxicity is also a problem in industrialized countries where they are not only used by the poorer segments of the society, but also in the treatment of cystic fibrosis (Prayle et al., 2016), in renal dialysis (Sowinski et al., 2008), and in emergencies. The World Health Organization recommends the use of aminoglycosides as part of the treatment against multidrug resistant tuberculosis (WHO, 2005).

It is generally accepted that, *in vivo*, aminoglycosides predominantly cross the BLB into the stria vascularis and, from there via marginal cells, into the endolymph (Li and Steyger, 2011). Once in the endolymph, these drugs would rapidly enter cochlear hair cells via mechanoelectrical transduction channels located on the stereocilia hair bundle, at their apical pole, and induce hair cell death (Marcotti et al., 2005; Alharazneh et al., 2011; Li and Steyger, 2011). Importantly, recent results suggest that inflammation boosts BLB permeability to aminoglycoside antibiotics, increasing the probability of drug-induced hearing loss (Koo et al., 2015). A number of studies in animal models, supported by studies *in vitro*, have established that ROS participate in the etiology of aminoglycoside-induced hearing loss (Forge and Schacht, 2000). From a clinical perspective, the more appealing result in support of this idea is the significant attenuation of gentamicin ototoxicity in humans by concurrent administration of aspirin detected in prospective, randomized, double-blind trials (Chen et al., 2007; Behnoud et al., 2009).

Another agent with recognized protective effects against aminoglycosides ototoxicity is the IGF1, a protein known to control cell proliferation, differentiation and apoptosis in various tissues and organs (Varela-Nieto et al., 2007). IGF1 is able to induce supporting cells in the mammalian organ of Corti to release the pro-resolution mediator netrin-1, which binds to one of its receptors (UNC5B) expressed on sensory hair cells and inhibits aminoglycoside-provoked apoptosis (Yamahara et al., 2017). Importantly, the efficacy of IGF1 in treating idiopathic sudden sensorineural hearing loss in humans has been confirmed in clinical trials (Nakagawa et al., 2010, 2014).

Based on the aforementioned studies, it is apparent that aminoglycoside-induced toxicity involves high oxidative stress and associated pathological signaling mechanisms like modulation of pro- and anti-apoptotic cell responses (Jiang et al., 2005). Thus, agents having strong antioxidant properties may have the ability to halt aminoglycosides’ toxicity. However, as we discuss below, the use of anti-oxidants is a double-edge sword because ROS are important signaling molecules and intermediaries in triggering specific anti-inflammatory responses. The efficacy of aspirin and netrin-1, on the other hand, suggest that pro-resolving therapies could be the answer to prevention and/or amelioration of aminoglycoside ototoxicity.

### Resolution of Inflammation and Noise-Related Hearing Loss

Although noise-related hearing loss (NRHL) remains associated with oxidative stress (Haase and Prasad, 2016), strong evidence suggest that inflammation is also a major contributor to this disorder. Several studies have demonstrated inflammatory responses in the cochlea following exposure to traumatic noise involving up-regulation of pro-inflammatory mediators and rapid recruitment of inflammatory cells from the vascular system (Derebery, 1996; Hirose et al., 2005; Fujioka et al., 2006; Tornabene et al., 2006; Tan et al., 2008; Wakabayashi et al., 2010). Within mere hours following acoustic overstimulation, leukocytes from the lateral wall and spiral limbus infiltrate the SV and the ST (Hirose et al., 2005; Sautter et al., 2006; Tornabene et al., 2006; Wakabayashi et al., 2010; Du et al., 2011), the SV side of the Reissner’s membrane (Sautter et al., 2006), and the ST side of the basilar membrane (Tornabene et al., 2006; Yang et al., 2015; Figure 8); importantly, no phagocytic cells are usually found in the SM (Hirose et al., 2005; Sautter et al., 2006; Tornabene et al., 2006; Miyao et al., 2008; Du et al., 2011).

Several inflammation-related genes and proteins have been implicated in the cochlear response to noise (Fujioka et al., 2006; Kirkegaard et al., 2006; Tornabene et al., 2006; Shi and Nuttall, 2007; Yamamoto et al., 2009; Wakabayashi et al., 2010; Gratton et al., 2011; Nakamoto et al., 2012), yet the precise molecular mechanisms and the role of inflammation in the development of cochlear injury remain to be elucidated. One recent study reports an early increased expression and a latter peak of pro-inflammatory mediators in mice exposed to acute traumatic noise (Tan et al., 2016). The first peak was associated by the authors with the recruitment of inflammatory cells into the cochlea, whereas the second was related to reparative processes in response to cochlear damage. Chronic environmental noise exposure has also been linked to inflammatory processes in the cochlea (Tan et al., 2016). Interestingly, it was recently reported that a variable number of OHCs die immediately after exposure, while IHCs initially die in much smaller numbers but their death is spread out over days to months after noise exposure. Sometimes, noise damages supporting cells before sensory hair cells, and they may continue to degenerate for months after noise exposure (Bohne et al., 2017). It has been proposed that this delayed death of auditory cells is associated with inflammatory responses.

Since NRHL is frequently a predictable form of hearing loss, prevention through therapeutic intervention is feasible, and reduction or fast resolution of inflammation has the potential to be effective. The TNF-α inhibitor etanercept has been shown to reduce noise-induced threshold shifts in animals (Wang et al., 2003). Similarly, it was found that an anti-IL-6-receptor antibody protected mice from NRHL (Wakabayashi et al., 2010). The anti-inflammatory and pro-resolution glucocorticoid dexamethasone (DEXA), delivered to the round window membrane, has also been shown to reduce hearing loss in patients after noise exposure (Zhou et al., 2013; Harrop-Jones et al., 2016).

Recent animal experiments have shown that noise exposure can lead to the degeneration of specific subsets of the nerve terminals in the ear without affecting thresholds (Kujawa and Liberman, 2009). This loss in synaptic ribbons, which could be the primary initial event in the degenerative cascade observed after noise, has been termed cochlear synaptopathy (Hickox et al., 2017; Liberman and Kujawa, 2017). Importantly, in addition to noise exposure, cochlear synaptopathy has also been associated with both aging (Sergeyenko et al., 2013; Altschuler et al., 2015; Möhrle et al., 2016) and the administration of ototoxic drugs (Bourien et al., 2014; Li et al., 2016). The confirmation of the presence of synaptopathy in human populations, and its potential association with inflammatory mechanisms, is currently under investigation (Hickox et al., 2017).

### Resolution of Inflammation and Age-Related Hearing Loss

Although ARHL (*aka* presbycusis) is the most common form of hearing loss in adults, their cellular and molecular mechanisms are still poorly understood (Huang and Tang, 2010). ARHL is variably expressed, with large differences in hearing threshold levels and hearing disability between individuals (Davis, 1989). It is currently accepted that this variability is due to a combination of environmental and genetic factors (Uchida et al., 2011), further complicated by association with other forms of age-related morbidity including cardiovascular diseases (Hutchinson et al., 2010; Karpa et al., 2010), and dementia (Lin et al., 2011).

Another key contributor to several age-related diseases, including ARHL, is the state of chronic inflammation in the elderly known as “inflammaging” (Capri et al., 2006; Hunt et al., 2010; Leng et al., 2011; Baylis et al., 2013; Verschuur et al., 2014). Inflammaging is a consequence of immune-senescence, the aging of the immune system (Capri et al., 2006; Hunt et al., 2010). A potential link with inflammaging may be very important for ARHL, providing new approaches to prevent the development of this condition.

As a matter of fact, ARHL severity has already been linked to some factors associated with inflammation and inflammaging (Gates et al., 1993; Gates and Mills, 2005; Frisina et al., 2006; Verschuur et al., 2014). For instance, it has been shown that spiral ganglion cell damage can be caused by changes in the immune system (Iwai et al., 2003, 2008), while vascular and metabolic changes may affect the stria vascularis and, indirectly, cause inflammatory damage (Saitoh et al., 1995; Ohlemiller, 2009; Fetoni et al., 2011). In a clinical trial an association was found between serum immunoglobulin G and hearing loss in individuals over 60 years of age (Lasisi et al., 2011). Thus, there is a high probability that inflammation and inflammaging could play a role in ARHL.

Studies in a mouse model of age-related sensory cell degeneration showed four major findings (Frye et al., 2017). First, it is mature, fully differentiated tissue macrophages that are the major type of macrophage populations responsible for the cochlear immune response in ARHL, and newly infiltrated monocytes are rare. Second, the mature tissue macrophages display a site-dependent change in their morphology and numbers and these changes are related to the dynamic progression of sensory cell degeneration. Third, apical and basal macrophages display different phenotypes under steady state conditions and have different response patterns to sensory cell degeneration. Finally, mature tissue macrophages are a sensitive internal sensor for early sensory cell degeneration. Together, these results suggest that the macrophage-mediated immune response is an integral part of the cochlear response to age-related chronic sensory cell degeneration (Frye et al., 2017), and suggest that pro-resolution therapeutic intervention targeting macrophages could be important for ameliorating ARHL.

Importantly, a 3-year double-blind, randomized controlled trial, aimed at determining whether aspirin slows development or progression of ARHL, is currently being conducted in Australia (Lowthian et al., 2016).

## Current and Potential New Clinical Strategies

### Glucocorticoids

The glucocorticoid DEXA has been shown to protect auditory hair cells against inflammatory cytokines by activating cell survival pathways (Haake et al., 2009). In addition, DEXA would be able to suppress drug toxicity associated with the production of free radicals by up-regulating antioxidant enzyme activity (Himeno et al., 2002; Paksoy et al., 2011). It was suggested that a single intratympanic injection of a DEXA solution administered immediately prior to cisplatin treatment had an otoprotective effect in rats (Daldal et al., 2007), and repeated intratympanic injections provided significant otoprotection when initially administered at the time of cisplatin treatment (Hill et al., 2008). In the clinic, however, inconsistent responses are commonly observed due to variable and limited exposure with aqueous solutions (Bird et al., 2007, 2011). Since DEXA is cleared rapidly from the middle ear down the Eustachian tube, it was suggested that a better clinical efficacy could be achieved by maintaining therapeutic drug levels for prolonged periods of time (Fernandez et al., 2016). It was recently reported that using a hydrogel containing DEXA (OTO-104) facilitates the presence of DEXA at therapeutic levels in the inner ear compartment for weeks to months in guinea pigs and sheep (Wang et al., 2009; Piu et al., 2011), and that a single intratympanic injection of 6% OTO-104 almost completely protected guinea pigs from cisplatin ototoxicity (Fernandez et al., 2016).

As noted before, DEXA is known to work, at least in part, through the stimulation of synthesis and release of the pro-resolution mediator ANXA1. Thus, pharmacologic strategies based on ANXA1 or its synthetic peptides could be as effective as DEXA without its negative side effects.

### Anti-Oxidants

Oxidative stress, a condition characterized by intracellular levels of ROS that impair the work of lipids, proteins and DNA, has been linked to many inner ear pathologies. Therefore, the use of anti-oxidants to diminish ROS levels appears as a no-brainer clinical strategy to protect the auditory function. However, ROS also work as mediators of many important physiological functions in a process termed redox biology (D’Autréaux and Toledano, 2007; Finkel, 2011; Schieber and Chandel, 2014). For example, mammalian cells respond to ROS by activating metabolic pathways that either provide cell stress protection or trigger cell apoptosis as a clearance mechanism for damaged tissues (Chen et al., 2009; Schieber and Chandel, 2014). Thus, ROS have two faces: redox biology, where ROS activate signaling pathways to initiate physiological processes, and oxidative stress where excessive amounts of intracellular ROS may lead to cell damage or death (Finkel, 2011; Schieber and Chandel, 2014). A critical point is that “low”, “right”, or “excessive” levels of ROS are not only cell- and tissue-dependent, but they are also associated with the physiological condition of the whole organism at the time of the pharmacological intervention. For instance, the balance between ROS and antioxidant defenses change during the reproductive process in mammals, with the high ROS levels required for appropriate fertilization, embryonic implantation, embryogenesis and placental development (Al-Gubory et al., 2010; Leghi and Muhlhausler, 2016) decreasing later on to diminish the risk of pregnancy disorders, including first trimester miscarriage (Jenkins et al., 2004), preeclampsia (D’Souza et al., 2016) and intrauterine growth restriction (Scifres and Nelson, 2009), associated with the excessive formation of reactive free radicals. In inflammation, significant evidence suggest that ROS are essential second messengers in innate and adaptive immune cells (West et al., 2011; Kamiński et al., 2013), but high levels of ROS aggravate inflammatory responses, resulting in tissue damage and different pathologies (Mittal et al., 2014). Therefore, while excessive levels of ROS are often directly responsible for cell death, their complete neutralization with antioxidant agents may be counterproductive by preventing the activation of natural cell defense mechanisms (Schieber and Chandel, 2014).

The association of ROS to cell and tissue protection mechanisms, such as optimal pathogen clearance, suggest that antioxidants should not be administered in healthy individuals that have a robust anti-oxidant defense and a healthy immune system. In addition, the timing of antioxidant treatment is crucial. This is certainly the case with patients in the intensive care unit, with multiple clinical trials consistently showing no efficacy or even increasing mortality in patients with critical illness that have been treated with antioxidants (Szakmany et al., 2012). Moreover, since different immune cell’s subsets seems to have differential responses to ROS, it might be beneficial in the amelioration of cisplatin effects to increase a particular subset of T cells or macrophages by either increasing or decreasing ROS levels (Schieber and Chandel, 2014).

### Aspirin

As already mentioned aspirin is a unique drug with, among others, anti-inflammatory, pro-resolution and anti-oxidant properties. Importantly, aspirin can work in combination with other drugs inducing a synergic effect. For example, it has been shown that aspirin has the potential to reduce the severity of cisplatin-induced side effects related to hearing and balance, by inducing several anti-inflammatory cytokines (Grilli et al., 1996; Yin et al., 1998). Thus, a combination of cisplatin and aspirin is an attractive strategy for managing solid tumors whilst protecting the auditory system. However, major obstacles in administering free-drug formulations include the definitive exposure to the targets of interest, individual pharmacokinetics and bio-distribution parameters. These factors are extremely difficult to control when drugs are individually administered. Recently, single pro-drugs containing a drug combination that can potentially overcome these challenges have been generated (Pathak et al., 2014). It is expected that a cisplatin + aspirin treatment in the form of a single pro-drug might increase efficiency and reduce ototoxic side effects of chemotherapy. A similar approach could be valid for reducing aminoglycosides ototoxicity, since aspirin is the only drug to date that has showed beneficial effect in clinical trials (Sha et al., 2006; Behnoud et al., 2009).

### Adenosine

A role for adenosine in auditory function was suggested by experiments in frogs several decades ago (Bryant et al., 1987). Subsequent studies in chinchilla cochleae provided evidence that administration to rats of *R*-phenylisopropyladenosine (*R*-PIA), an agonist of the purinergic adenosine receptor A1AR, increased the activity of antioxidant enzymes and reduced lipid peroxidation (Ford et al., 1997a), while cisplatin exposure significantly increases the expression of adenosine receptors (Ford et al., 1997b). Studies in rats have also shown that *R*PIA protects cochlear explants from damage induced by cisplatin and noise (Hu et al., 1997; Hight et al., 2003). A potential role for adenosine in cochlear protection has been substantiated by more recent studies. For example, it has been shown that administration of adenosine amine congeners (ADAC) protect against noise-induced hearing loss (Vlajkovic et al., 2010), and elevation of adenosine levels protect against ARHL by inhibition of adenosine kinase (Vlajkovic et al., 2011). Furthermore, experimental results suggest that A1AR ameliorates cisplatin ototoxicity by inhibiting the NOX3/STAT1-mediated inflammatory pathway (Kaur et al., 2016).

Thus, drugs that increase the concentration of endogenous adenosine or directly activate adenosine receptors could play a pivotal role in the protection of the organ of Corti against cisplatin cytotoxicity. Once more, mediators of inflammatory resolution could serve as ideal targets for otoprotective therapies.

## Concluding Remarks

Decades of intensive research have not delivered successful clinical strategies for preventing or ameliorating DRHL, NRHL or ARHL. Currently there is not a single specific, FDA-approved otoprotective agent. In addition, the most common therapeutic approaches, glucocorticoid and antioxidants, produce inconsistent results. Thus, we feel that an alternative research paradigm is absolutely needed to break the stalemate.

The typical research approach has been, and still is, to look for prevention of cell damage induced by the toxic agents under investigation. Although this approach is reasonable and absolutely valid, we want to propose an alternative way: enhance the physiological mechanisms designed for our organism for dealing with toxic agents, looking for an accelerated and improved cell and tissue protection, healing and repair. Specifically, we propose to select the pro-resolution pathways associated with the successful termination of inflammatory responses as a new target for research aimed at preventing or ameliorating DRHL, NRHL and ARHL. We strongly believe that improving the resolution of cochlear inflammatory responses is one way, although most likely not the only one, to overcome the current impasse in this important area of hearing research.

Inflammation is a beneficial host reaction aimed at protecting individuals from infections and tissue injury. Uncontrolled inflammation, however, is now widely recognized as a common factor in many diseases and organ dysfunctions, including DRHL, NRHL and ARHL. Since inflammatory responses are aimed to eliminate invading organisms and repair injured tissues, they are naturally self-limited. Resolution, as the last step in any inflammatory response, is exquisitely regulated, and it is completed only after any potential for continuous tissue damage has been conquered. Thus, improving the resolution phase of inflammatory responses in the inner ear may naturally result in cell protection, tissue healing and repair, therefore contributing to the prevention or amelioration of auditory dysfunctions.

The success of the proposed approach is heavily dependent on the full understanding of resolution biology and the expression and function of pro-resolution mediators and receptors in the mammalian cochlea. Unfortunately, the current number of studies on this topic in the inner ear is clearly insufficient. Therefore, we consider imperative to accelerate the identification of all pro-resolution pathways at work in the cochlea. Next, their specific functions should be explored to understand how to enhance, safely and rapidly, the resolution of inflammatory responses in the inner ear. Of course, we are not proposing to eliminate any of the current research strategies, since it is unlikely that any single approach will be the magic bullet for all non-resolving, chronic inflammatory and autoimmune diseases. Most likely combinatorial pro-resolving therapies also including, perhaps, dietary w-3 and w-6 essential fatty acids and inhibitors of specific pro-inflammatory agents and their receptors, would be necessary to obtain significant effects. Regardless, we are convinced that the elucidation of the mechanisms of pharmacological modulation of the resolution process would be crucial to finding the most effective therapeutic agents for preventing or ameliorating DRHL, NRHL and ARHL.

## Author Contributions

FK saw the need of a review on this topic. GMK and GL reviewed the literature and organized the information. RAU and FK wrote the manuscript.

## Conflict of Interest Statement

The authors declare that the research was conducted in the absence of any commercial or financial relationships that could be construed as a potential conflict of interest. The reviewer MJ and handling Editor declared their shared affiliation, and the handling Editor states that the process nevertheless met the standards of a fair and objective review.
